# Intraoperative Correction of Femoral Anteversion Using a Native Smartphone Measurement Tool

**DOI:** 10.7759/cureus.114550

**Published:** 2026-08-14

**Authors:** Parker K Chenault, Owen M Ball, Cody L Evans, Franco M Coniglione

**Affiliations:** 1 Orthopaedics, Virginia Tech Carilion School of Medicine, Roanoke, USA; 2 Orthopaedic Surgery, Carilion Clinic, Roanoke, USA

**Keywords:** derotation osteotomy, internal fixation, non-union shaft femur, novel surgical technique, trauma and orthopedic surgery

## Abstract

Femoral de-rotation osteotomy is an established treatment option for femoral malrotation. Surgical correction of femoral malrotation often requires precise measurements using specialized equipment. Identification of a widely accessible intraoperative measurement tool that may be used in place of this specialized equipment may improve surgeons' accuracy with regard to achieving the desired degree of femoral rotation and therefore improve postoperative outcomes. In this case report, we highlight the use of a widely available smartphone application to measure and operatively correct femoral malrotation.

## Introduction

Femoral version, defined as the angle created between the axis of the femoral neck and the transcondylar axis of the knee, has a normal range between 10-25 degrees of anteversion [1,2]. Excessive anteversion of the femur may result in in-toeing, bow-leggedness, pain in the hips, knees, and ankles, or a snapping sound in the hip while walking ​[3]. Femoral de-rotation osteotomy using intramedullary nail fixation is an established method of surgical treatment for symptomatic, excessive anteversion of the femur [4]. Currently, there is a large degree of variation with regard to intraoperative measurement techniques during de-rotation osteotomy. Previously described de-rotation techniques include making a transverse cut in the shaft of the femur, flexing the hip to 90 degrees, and internally or externally rotating the hip to the point of impingement. Using this method, visual estimates of the desired degree of internal or external rotation are used to make corrections to rotation [5]​​. Alternatively, Mayne et al. described the use of a bubble inclinometer to provide an objective measurement of rotation of the femur [6]​. Previous studies using sawbones models have shown that measurements taken using a native smartphone application are significantly more accurate than either visual estimation or osteotomy templates [7]. This paper describes a technique in which a widely available smartphone application was used to measure and adjust femoral rotation during a de-rotation surgery.     

This article was submitted to the American Academy of Orthopedic Surgeons 2026 annual conference.

## Case presentation

**Figure 1:**
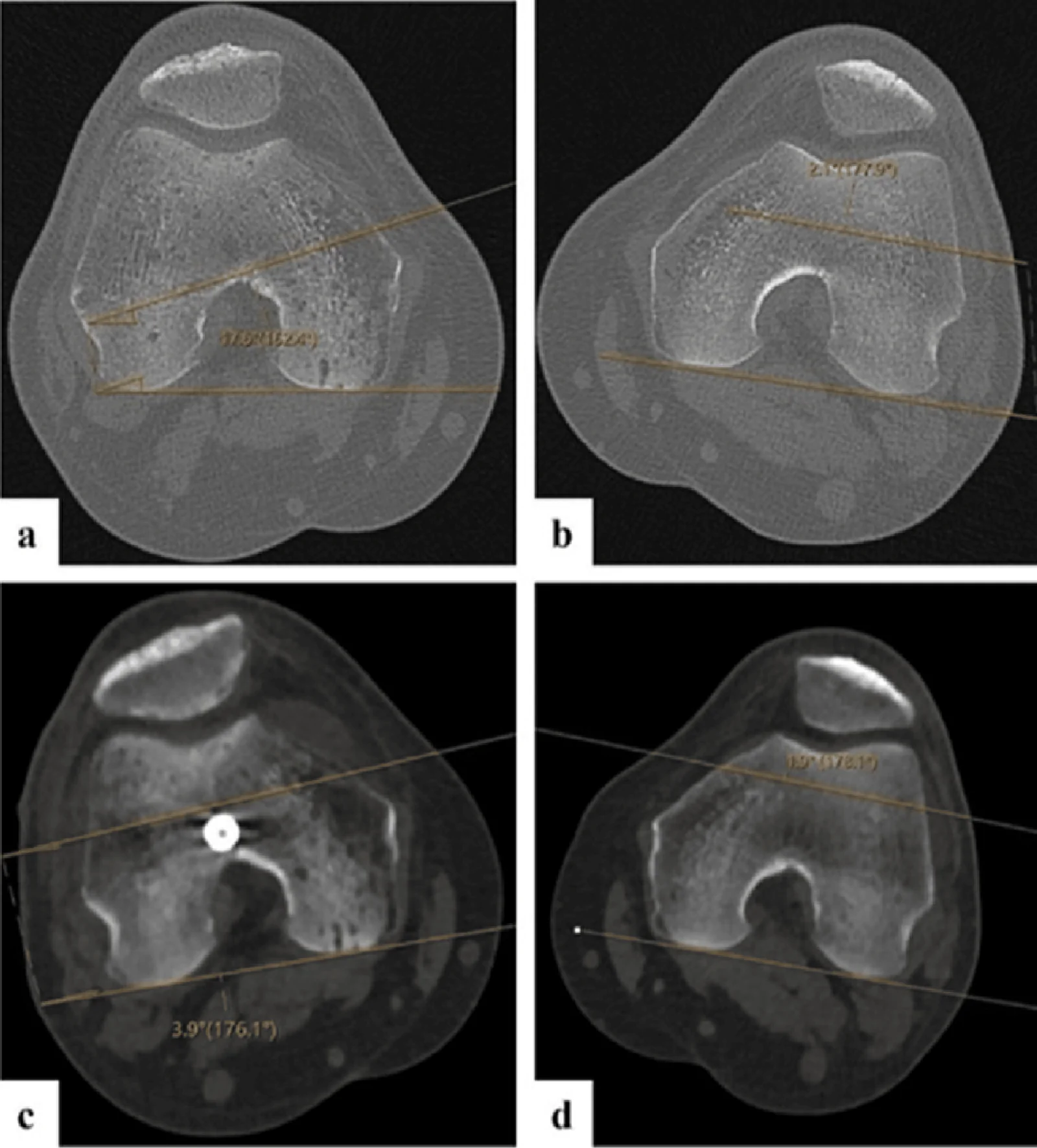
Computed Tomography Scanogram Non-contrast CT was used to determine femoral anteversion of both affected and unaffected lower extremities. a) Preoperative CT of the affected extremity shows a femoral anteversion of 17.6° b) Preoperative CT of the unaffected extremity shows a femoral anteversion of 2.1° c) Postoperative CT of the affected extremity shows a femoral anteversion of 3.9° d) Postoperative CT of the unaffected extremity showing a femoral anteversion of 1.9°.

A 41-year-old male presented with persistent right leg weakness, thigh pain, and difficulty ambulating six months post fixation of a right comminuted mid-shaft femur fracture that was treated surgically via intramedullary nailing. Radiographs revealed stable hardware with no signs of failure, persistent non-union of the fracture, and a rotational deformity at the fracture site. A computed tomography (CT) scanogram of the bilateral femora was obtained. Scans were reviewed and measured by an independent radiologist, revealing a difference of 15.5 degrees between the two sides (Figure 1).

**Figure 2:**
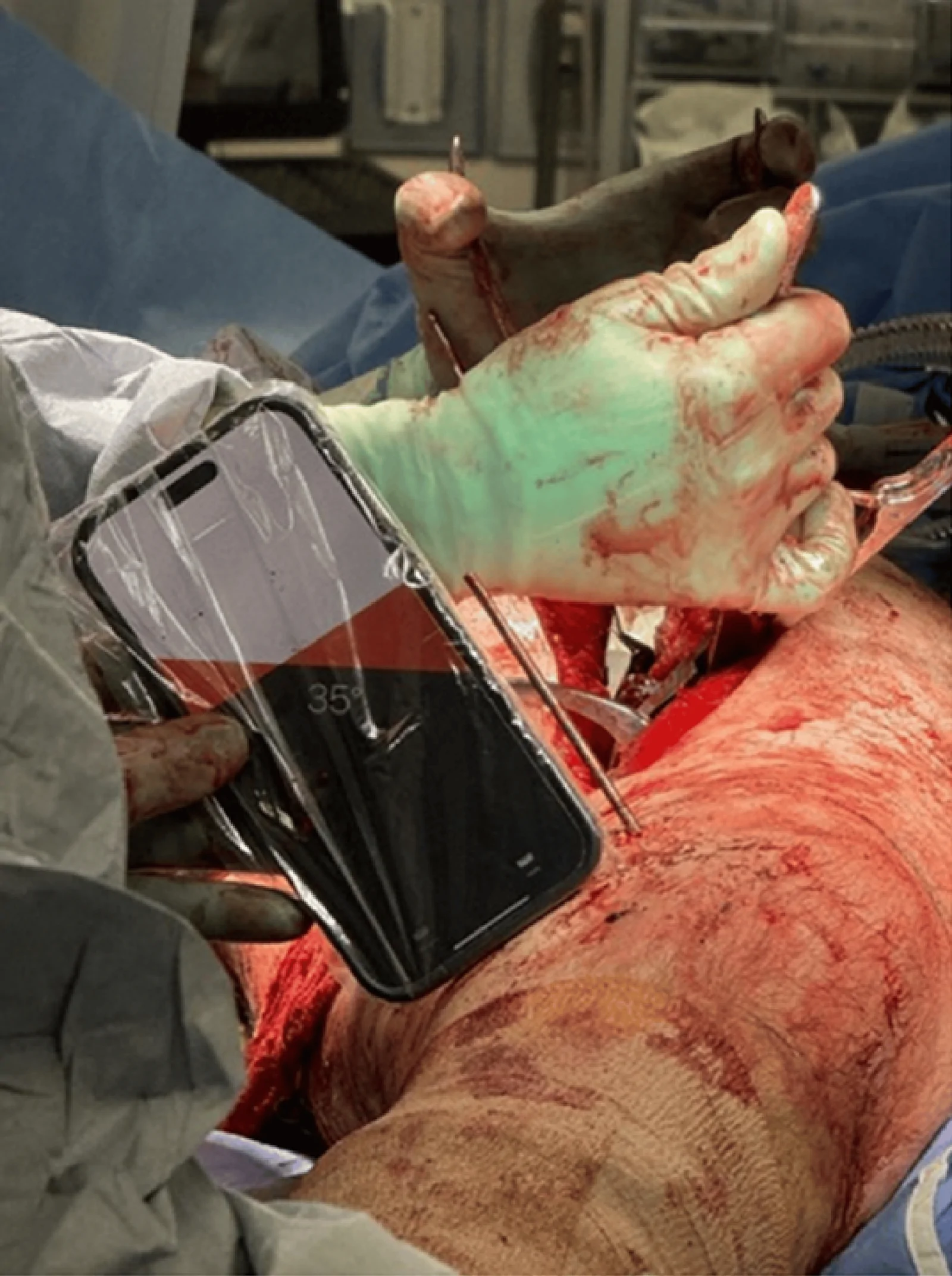
Measurement Method The “Level” function within the native "Measure" app on the smartphone was used to assess the anteversion of the femur intraoperatively. Anteversion was determined by placing the smartphone parallel to the fixed Schanz screws and subtracting the value of the distal screw from the proximal screw. Here, the distal Shanz screw is shown inserted along the femoral condylar axis while the surgeon rotates the femur proximally using bone reduction forceps.

The patient was scheduled for a right femoral de-rotation osteotomy for correction of the rotational deformity. Intraoperatively, Shanz screws were inserted into the proximal and distal segments to use as reference points. A smartphone (iPhone 16 Pro Max, Apple, Cupertino, CA, USA) was placed in a sterilized bag, and the long edge of the device was positioned flush against the Shanz screws. The “Measure” application was utilized to obtain angular measurements of the individual proximal and distal screws (Figure 2).

**Figure 3:**
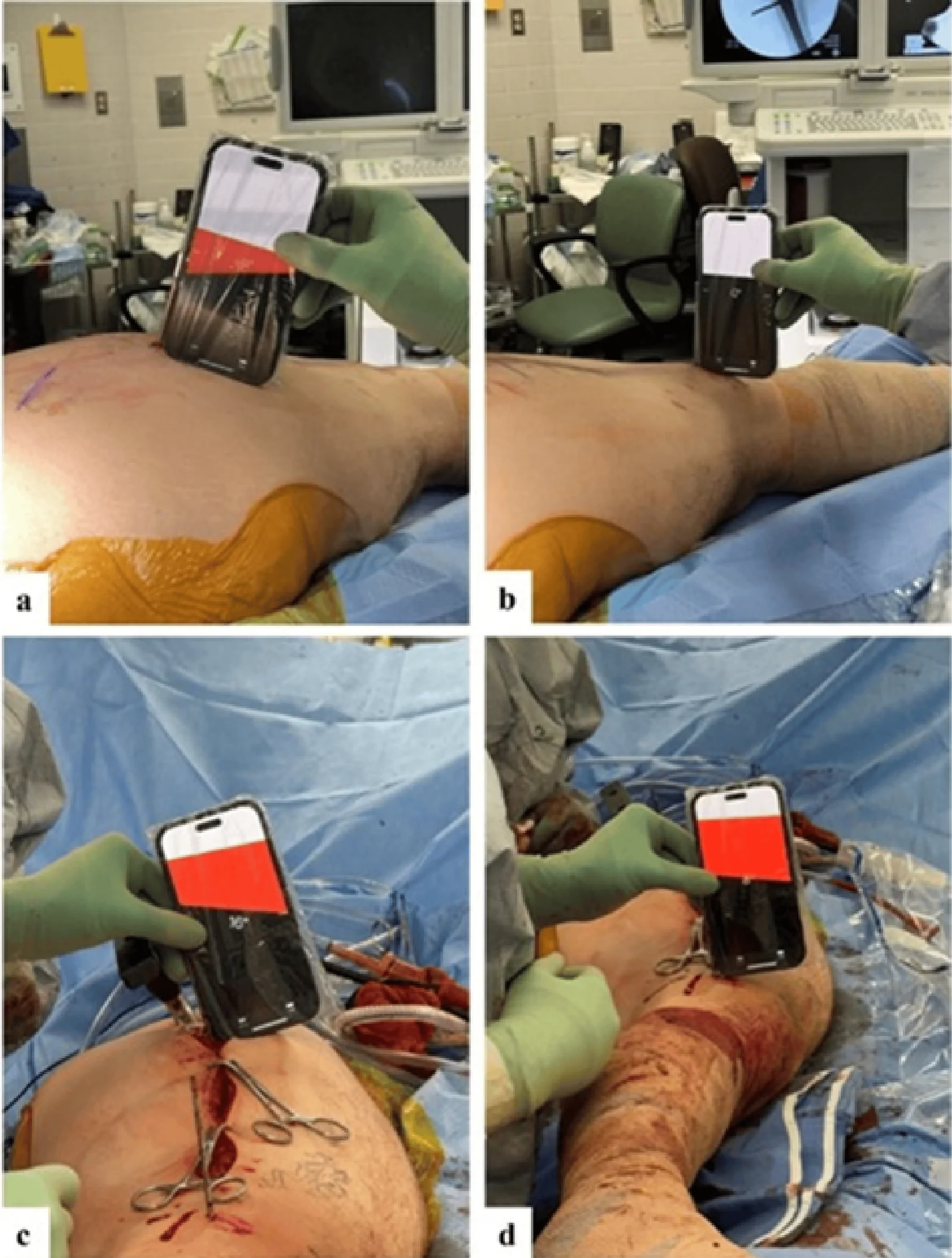
Preoperative and Postoperative Measurements Anteversion of 18° and 8° were assessed preoperatively and postoperatively, respectively, by subtracting the value read from the distal screw from the value read at the proximal screw. a) Preoperative proximal screw assessment yielded a value of 0° b) Preoperative distal screw assessment yielded a value of -18° c) Postoperative proximal screw assessment yielded a value of 16° d) Postoperative distal screw assessment yielded a value of 8°.

The relative angular difference between the two screws was then calculated to be 18 degrees. Given the preoperative interfemoral angular discrepancy of 15.5 degrees measured via CT, a relative angular difference measured via smartphone of 2.5 degrees was desired. In this case, given the presence of femoral nonunion (in addition to femoral malrotation), exchange nailing of the femur was indicated to promote healing. The original intramedullary implant was removed. Rotational correction was performed using the rigid Schanz screws as joysticks with confirmation using the aforementioned app, followed by provisional clamp reduction of the fracture. Reaming of the canal was performed, and a new intramedullary implant was inserted. The implant was locked into the head/neck segment with recon screws. Rotation of the femur was corrected through the open exposure. The desired correction amount was verified with the use of the Schanz screws and smartphone app (Figure 3).

Once the correction was confirmed, the implant was locked distally with interlocking screws. A postoperative CT scan was performed to measure the actual correction achieved. The preoperative anteversion of the unaffected femur was 2.1 degrees as compared to 17.6 degrees on the operative side. Postoperatively, the corrected anteversion of the affected femur was measured as 3.9 degrees and the unaffected femur had a measured anteversion of 1.9 degrees, a decrease in anteversion of 13.5 degrees and within the target range of 2 degrees of the unaffected side. The actual change in anteversion was 87% of the desired change. 

## Discussion

This case successfully highlights the use of a widely available smartphone application to measure and correct femoral malrotation. This technique may offer several advantages over previous techniques. The use of this tool allows for objective measurement of the rotation of the femur intraoperatively and would likely be superior to previously described techniques using visual estimation to achieve the desired degree of femoral rotation. Additionally, other objective measurement tools like inclinometers are not widely available and the native smartphone measurement tool is nearly universally accessible. The implementation of this method would also be cost-effective as it would not require the purchase of any additional surgical equipment.

With the average difference in anteversion of the femurs in a given person being only five degrees, it is important to achieve the highest degree of accuracy possible when performing a de-rotation osteotomy [8]​​. By achieving a corrected anteversion of the femur within two degrees of the contralateral side, this case report demonstrates that a high degree of accuracy with regard to femoral rotation is achievable using the native smartphone measurement tool. 

This method of femoral rotation measurement may be limited by unintended movement of the Schanz screws during the operation which may impact the accuracy of measurements taken once the distal interlocking screw is placed. This may explain the discrepancy between the change in anteversion measured intraoperatively compared to the change calculated using preoperative and postoperative CT scans. However, this discrepancy may indicate a limitation with regard to repeatability of measurements taken with the smartphone application. Additionally, while the phone is placed in a sterile bag, this method could potentially introduce contaminants into the surgical field. As such, standardized protocols for the use of smartphones intraoperatively may be necessary to minimize the risk of contamination.

## Conclusions

This case report demonstrates that the use of a native smartphone app as an intraoperative measurement tool is a feasible technique for achieving appropriate rotational correction of a femur. A widely available intraoperative measurement tool may help increase accessibility and accuracy with regard to achieving the desired rotation of a femur when performing a de-rotation osteotomy. While intraoperative measurement tools may not be available in many operating rooms, smartphones are widely accessible and are a potentially useful tool for measuring and verifying changes in femoral rotation.
